# Supplementary material for: High-Throughput Fingerprinting of Rhizobial Free Fatty Acids by Chemical Thin-Film Deposition and Matrix-Assisted Laser Desorption/Ionization Mass Spectrometry
Source: Methods Protoc. 2020 May 4;3(2):36. doi: 10.3390/mps3020036 (PMC7359708; doi:10.3390/mps3020036)

Bacteria_Autoxecute_hexane_normalization by 421.17

Experiment Name: Bacteria_Autoxecute_hexane

Experiment Design

Group	Rhizobium leguminosarum bv. viciae RCAM1026	Sinorhizobium meloloti RCAM1021
Replicates	4	4

Spectra	Sample	Group
Rh.leg.RCAM2016_1-1-1	Rhizobium leguminosarum bv. viciae RCAM1026_1	Rhizobium leguminosarum bv. viciae RCAM1026
Rh.leg.RCAM2016_1-1-2	Rhizobium leguminosarum bv. viciae RCAM1026_1	Rhizobium leguminosarum bv. viciae RCAM1026
Rh.leg.RCAM2016_1-1-3	Rhizobium leguminosarum bv. viciae RCAM1026_1	Rhizobium leguminosarum bv. viciae RCAM1026
Rh.leg.RCAM2016_1-2-1	Rhizobium leguminosarum bv. viciae RCAM1026_1	Rhizobium leguminosarum bv. viciae RCAM1026
Rh.leg.RCAM2016_1-2-2	Rhizobium leguminosarum bv. viciae RCAM1026_1	Rhizobium leguminosarum bv. viciae RCAM1026
Rh.leg.RCAM2016_1-2-3	Rhizobium leguminosarum bv. viciae RCAM1026_1	Rhizobium leguminosarum bv. viciae RCAM1026
Rh.leg.RCAM2016_1-3-1	Rhizobium leguminosarum bv. viciae RCAM1026_1	Rhizobium leguminosarum bv. viciae RCAM1026
Rh.leg.RCAM2016_1-3-2	Rhizobium leguminosarum bv. viciae RCAM1026_1	Rhizobium leguminosarum bv. viciae RCAM1026
Rh.leg.RCAM2016_1-3-3	Rhizobium leguminosarum bv. viciae RCAM1026_1	Rhizobium leguminosarum bv. viciae RCAM1026
Rh.leg.RCAM2016_2-1-1	Rhizobium leguminosarum bv. viciae RCAM1026_2	Rhizobium leguminosarum bv. viciae RCAM1026
Rh.leg.RCAM2016_2-1-2	Rhizobium leguminosarum bv. viciae RCAM1026_2	Rhizobium leguminosarum bv. viciae RCAM1026
Rh.leg.RCAM2016_2-1-3	Rhizobium leguminosarum bv. viciae RCAM1026_2	Rhizobium leguminosarum bv. viciae RCAM1026
Rh.leg.RCAM2016_2-2-1	Rhizobium leguminosarum bv. viciae RCAM1026_2	Rhizobium leguminosarum bv. viciae RCAM1026
Rh.leg.RCAM2016_2-2-2	Rhizobium leguminosarum bv. viciae RCAM1026_2	Rhizobium leguminosarum bv. viciae RCAM1026
Rh.leg.RCAM2016_2-2-3	Rhizobium leguminosarum bv. viciae RCAM1026_2	Rhizobium leguminosarum bv. viciae RCAM1026
Rh.leg.RCAM2016_2-3-1	Rhizobium leguminosarum bv. viciae RCAM1026_2	Rhizobium leguminosarum bv. viciae RCAM1026
Rh.leg.RCAM2016_2-3-2	Rhizobium leguminosarum bv. viciae RCAM1026_2	Rhizobium leguminosarum bv. viciae RCAM1026
Rh.leg.RCAM2016_2-3-3	Rhizobium leguminosarum bv. viciae RCAM1026_2	Rhizobium leguminosarum bv. viciae RCAM1026
Rh.leg.RCAM2016_3-1-1	Rhizobium leguminosarum bv. viciae RCAM1026_3	Rhizobium leguminosarum bv. viciae RCAM1026
Rh.leg.RCAM2016_3-1-2	Rhizobium leguminosarum bv. viciae RCAM1026_3	Rhizobium leguminosarum bv. viciae RCAM1026
Rh.leg.RCAM2016_3-1-3	Rhizobium leguminosarum bv. viciae RCAM1026_3	Rhizobium leguminosarum bv. viciae RCAM1026
Rh.leg.RCAM2016_3-2-1	Rhizobium leguminosarum bv. viciae RCAM1026_3	Rhizobium leguminosarum bv. viciae RCAM1026
Rh.leg.RCAM2016_3-2-2	Rhizobium leguminosarum bv. viciae RCAM1026_3	Rhizobium leguminosarum bv. viciae RCAM1026
Rh.leg.RCAM2016_3-2-3	Rhizobium leguminosarum bv. viciae RCAM1026_3	Rhizobium leguminosarum bv. viciae RCAM1026
Rh.leg.RCAM2016_3-3-1	Rhizobium leguminosarum bv. viciae RCAM1026_3	Rhizobium leguminosarum bv. viciae RCAM1026
Rh.leg.RCAM2016_3-3-2	Rhizobium leguminosarum bv. viciae	Rhizobium leguminosarum bv. viciae

Spectra	Sample	Group
	RCAM1026_3	RCAM1026
Rh.leg.RCAM2016_3-3-3	Rhizobium leguminosarum bv. viciae RCAM1026_3	Rhizobium leguminosarum bv. viciae RCAM1026
Rh.leg.RCAM2016_4-1-1	Rhizobium leguminosarum bv. viciae RCAM1026_4	Rhizobium leguminosarum bv. viciae RCAM1026
Rh.leg.RCAM2016_4-1-2	Rhizobium leguminosarum bv. viciae RCAM1026_4	Rhizobium leguminosarum bv. viciae RCAM1026
Rh.leg.RCAM2016_4-1-3	Rhizobium leguminosarum bv. viciae RCAM1026_4	Rhizobium leguminosarum bv. viciae RCAM1026
Rh.leg.RCAM2016_4-2-1	Rhizobium leguminosarum bv. viciae RCAM1026_4	Rhizobium leguminosarum bv. viciae RCAM1026
Rh.leg.RCAM2016_4-2-2	Rhizobium leguminosarum bv. viciae RCAM1026_4	Rhizobium leguminosarum bv. viciae RCAM1026
Rh.leg.RCAM2016_4-2-3	Rhizobium leguminosarum bv. viciae RCAM1026_4	Rhizobium leguminosarum bv. viciae RCAM1026
Rh.leg.RCAM2016_4-3-1	Rhizobium leguminosarum bv. viciae RCAM1026_4	Rhizobium leguminosarum bv. viciae RCAM1026
Rh.leg.RCAM2016_4-3-2	Rhizobium leguminosarum bv. viciae RCAM1026_4	Rhizobium leguminosarum bv. viciae RCAM1026
Rh.leg.RCAM2016_4-3-3	Rhizobium leguminosarum bv. viciae RCAM1026_4	Rhizobium leguminosarum bv. viciae RCAM1026
Si.mel.RCAM1021_1-1-1	Sinorhizobium meloloti RCAM1021_1	Sinorhizobium meloloti RCAM1021
Si.mel.RCAM1021_1-1-2	Sinorhizobium meloloti RCAM1021_1	Sinorhizobium meloloti RCAM1021
Si.mel.RCAM1021_1-1-3	Sinorhizobium meloloti RCAM1021_1	Sinorhizobium meloloti RCAM1021
Si.mel.RCAM1021_1-2-1	Sinorhizobium meloloti RCAM1021_1	Sinorhizobium meloloti RCAM1021
Si.mel.RCAM1021_1-2-2	Sinorhizobium meloloti RCAM1021_1	Sinorhizobium meloloti RCAM1021
Si.mel.RCAM1021_1-2-3	Sinorhizobium meloloti RCAM1021_1	Sinorhizobium meloloti RCAM1021
Si.mel.RCAM1021_1-3-1	Sinorhizobium meloloti RCAM1021_1	Sinorhizobium meloloti RCAM1021
Si.mel.RCAM1021_1-3-2	Sinorhizobium meloloti RCAM1021_1	Sinorhizobium meloloti RCAM1021
Si.mel.RCAM1021_1-3-3	Sinorhizobium meloloti RCAM1021_1	Sinorhizobium meloloti RCAM1021
Si.mel.RCAM1021_2-1-1	Sinorhizobium meloloti RCAM1021_2	Sinorhizobium meloloti RCAM1021
Si.mel.RCAM1021_2-1-2	Sinorhizobium meloloti RCAM1021_2	Sinorhizobium meloloti RCAM1021
Si.mel.RCAM1021_2-1-3	Sinorhizobium meloloti RCAM1021_2	Sinorhizobium meloloti RCAM1021
Si.mel.RCAM1021_2-2-1	Sinorhizobium meloloti RCAM1021_2	Sinorhizobium meloloti RCAM1021
Si.mel.RCAM1021_2-2-2	Sinorhizobium meloloti RCAM1021_2	Sinorhizobium meloloti RCAM1021
Si.mel.RCAM1021_2-2-3	Sinorhizobium meloloti RCAM1021_2	Sinorhizobium meloloti RCAM1021
Si.mel.RCAM1021_2-3-1	Sinorhizobium meloloti RCAM1021_2	Sinorhizobium meloloti RCAM1021
Si.mel.RCAM1021_2-3-2	Sinorhizobium meloloti RCAM1021_2	Sinorhizobium meloloti RCAM1021
Si.mel.RCAM1021_2-3-3	Sinorhizobium meloloti RCAM1021_2	Sinorhizobium meloloti RCAM1021
Si.mel.RCAM1021_3-1-1	Sinorhizobium meloloti RCAM1021_3	Sinorhizobium meloloti RCAM1021
Si.mel.RCAM1021_3-1-2	Sinorhizobium meloloti RCAM1021_3	Sinorhizobium meloloti RCAM1021
Si.mel.RCAM1021_3-1-3	Sinorhizobium meloloti RCAM1021_3	Sinorhizobium meloloti RCAM1021
Si.mel.RCAM1021_3-2-1	Sinorhizobium meloloti RCAM1021_3	Sinorhizobium meloloti RCAM1021
Si.mel.RCAM1021_3-2-2	Sinorhizobium meloloti RCAM1021_3	Sinorhizobium meloloti RCAM1021
Si.mel.RCAM1021_3-2-3	Sinorhizobium meloloti RCAM1021_3	Sinorhizobium meloloti RCAM1021
Si.mel.RCAM1021_3-3-1	Sinorhizobium meloloti RCAM1021_3	Sinorhizobium meloloti RCAM1021
Si.mel.RCAM1021_3-3-2	Sinorhizobium meloloti RCAM1021_3	Sinorhizobium meloloti RCAM1021
Si.mel.RCAM1021_3-3-3	Sinorhizobium meloloti RCAM1021_3	Sinorhizobium meloloti RCAM1021
Si.mel.RCAM1021_4-1-1	Sinorhizobium meloloti RCAM1021_4	Sinorhizobium meloloti RCAM1021
Si.mel.RCAM1021_4-1-2	Sinorhizobium meloloti RCAM1021_4	Sinorhizobium meloloti RCAM1021
Si.mel.RCAM1021_4-1-3	Sinorhizobium meloloti RCAM1021_4	Sinorhizobium meloloti RCAM1021
Si.mel.RCAM1021_4-2-1	Sinorhizobium meloloti RCAM1021_4	Sinorhizobium meloloti RCAM1021
Si.mel.RCAM1021_4-2-2	Sinorhizobium meloloti RCAM1021_4	Sinorhizobium meloloti RCAM1021
Si.mel.RCAM1021_4-2-3	Sinorhizobium meloloti RCAM1021_4	Sinorhizobium meloloti RCAM1021
Si.mel.RCAM1021_4-3-1	Sinorhizobium meloloti RCAM1021_4	Sinorhizobium meloloti RCAM1021
Si.mel.RCAM1021_4-3-2	Sinorhizobium meloloti RCAM1021_4	Sinorhizobium meloloti RCAM1021
Si.mel.RCAM1021_4-3-3	Sinorhizobium meloloti RCAM1021_4	Sinorhizobium meloloti RCAM1021

Statistics Measurement: Normalized Peak Height

Normalization Method: Single Peak Height 421.17 m/z

Peaks

#	m/z	Fold	Anova (p)	Tags
3	363.09	1.08	0.0905	
5	365.11	1.07	0.133	
13	377.11	1.13	0.000964	
15	379.12	1.15	0.0225	
24	391.12	1.10	0.0219	
26	393.14	1.19	0.000927	
45	417.14	1.19	0.000232	
49	419.16	1.16	9.98E-06	
51	421.17	1.00	1	

Peaks : Average Normalized Peak Heights

#	Rhizobium leguminosarum bv. viciae RCAM1026	Sinorhizobium meloloti RCAM1021
3	806.282	744.536
5	2393.779	2230.531
13	1181.413	1046.856
15	3749.689	3250.885
24	1.528e+004	1.387e+004
26	2.628e+004	2.216e+004
45	1.568e+004	1.322e+004
49	4.911e+004	4.236e+004
51	9.700e+004	9.700e+004

Identifier 3: m/z 363.09

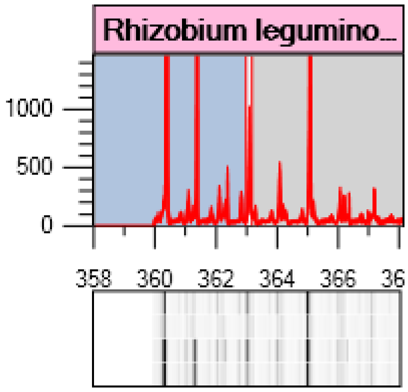

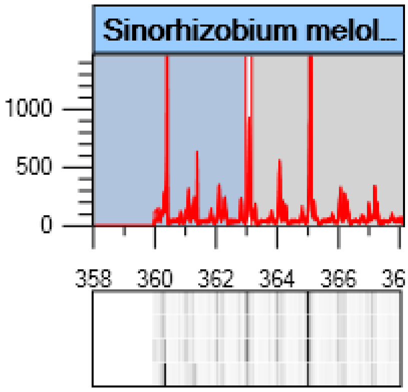

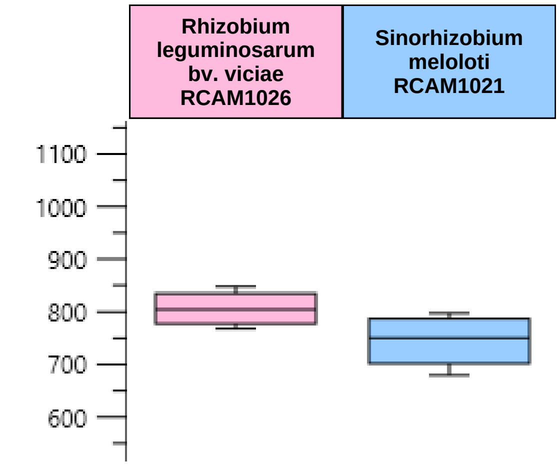

Identifier 5: m/z 365.11

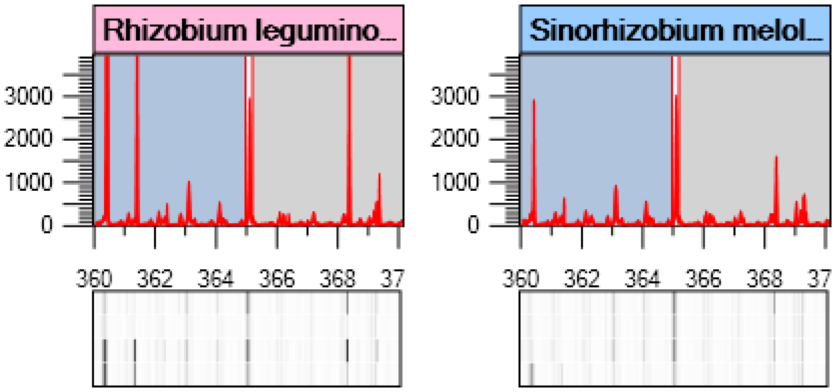

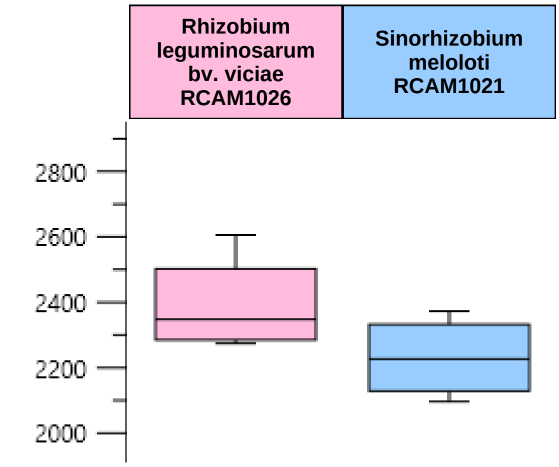

Identifier 13: m/z 377.11

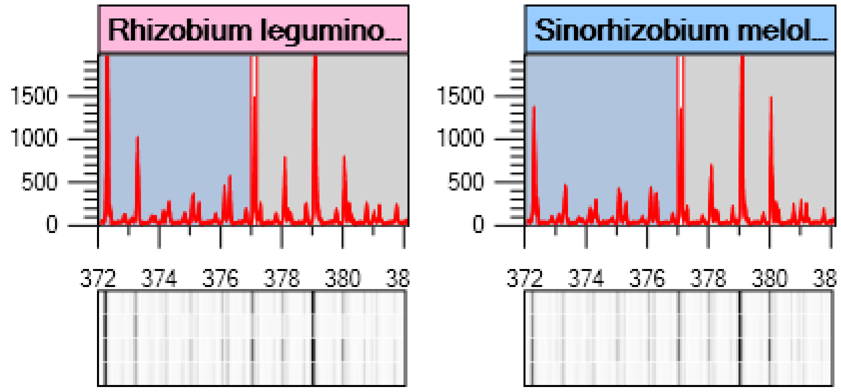

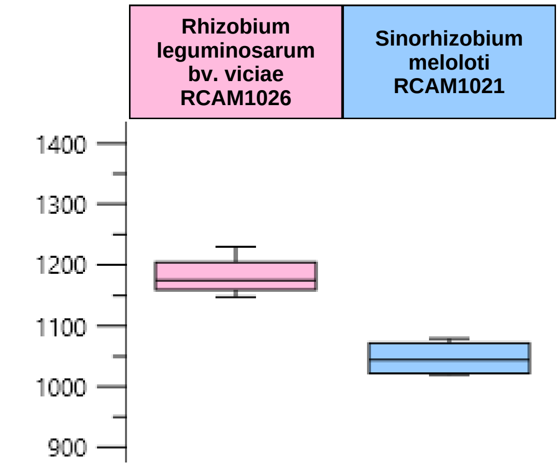

Identifier 15: m/z 379.12

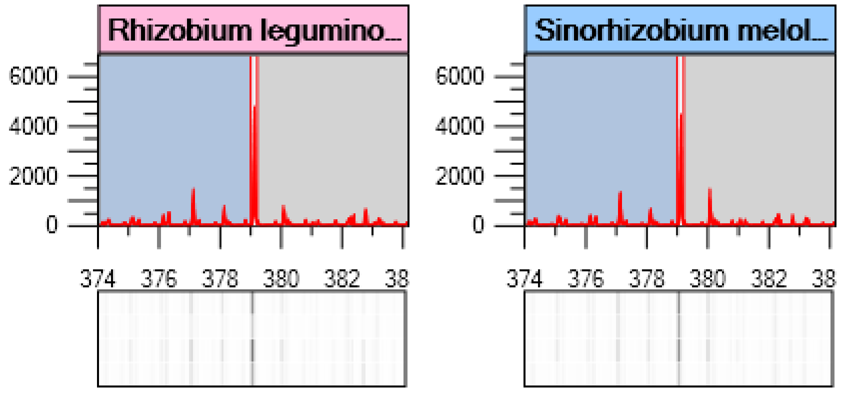

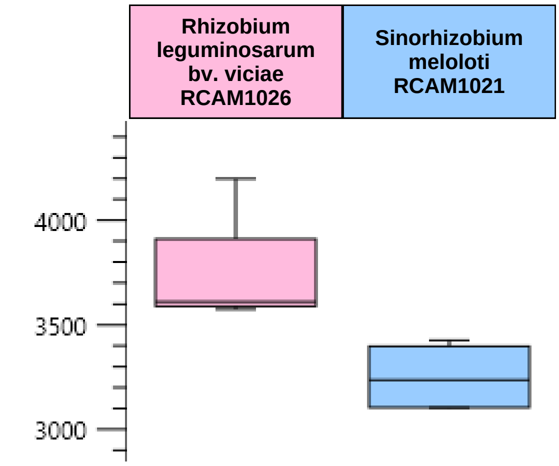

Identifier 24: m/z 391.12

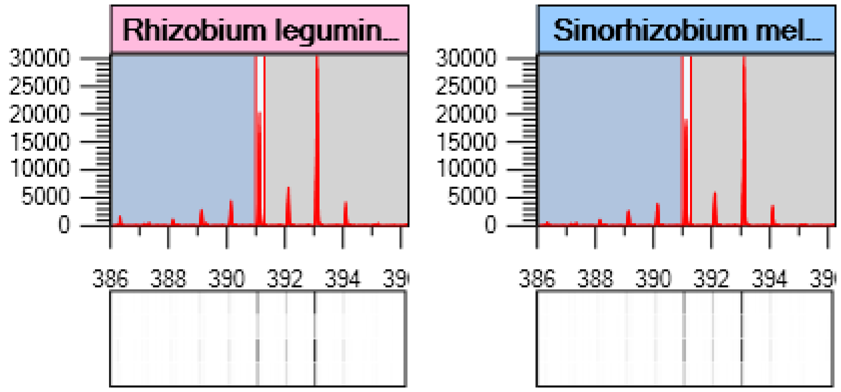

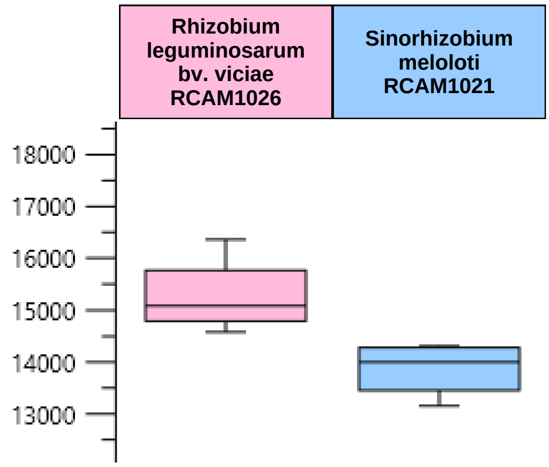

Identifier 26: m/z 393.14

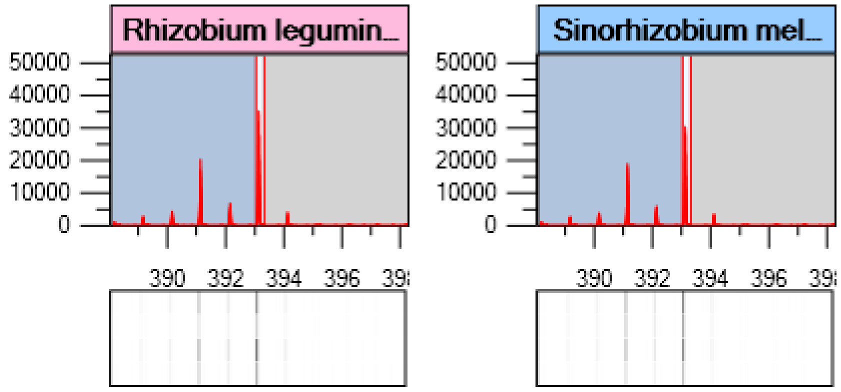

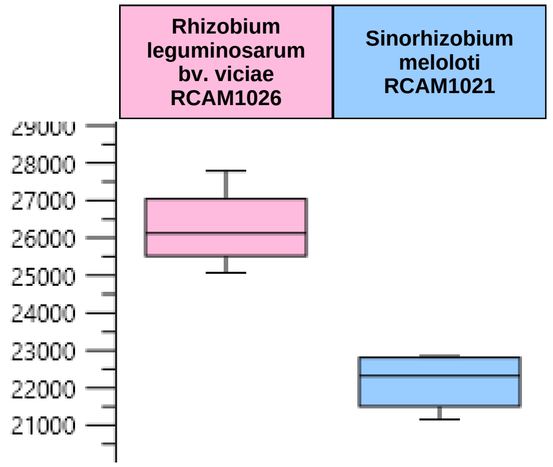

Identifier 45: m/z 417.14

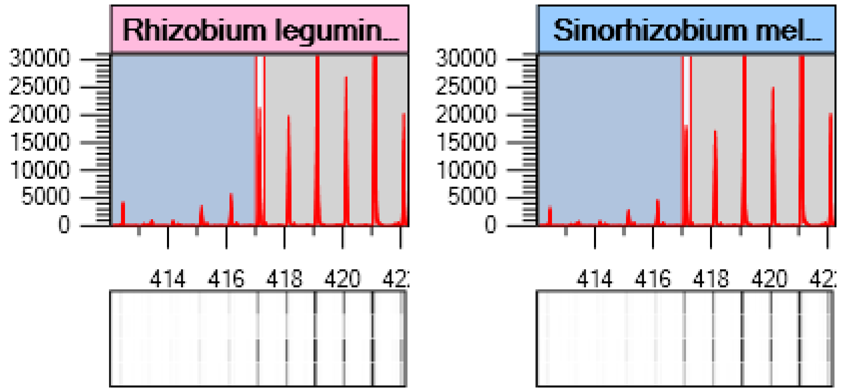

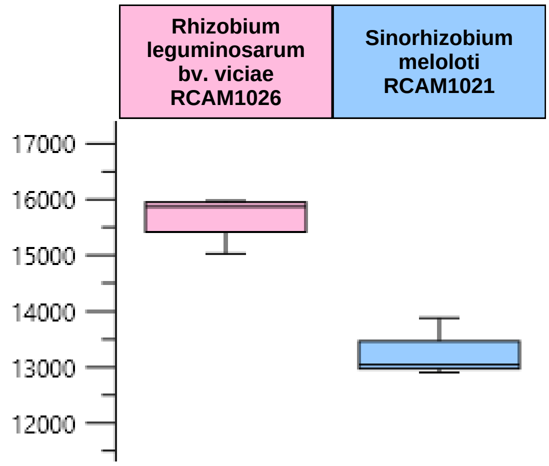

Identifier 49: m/z 419.16

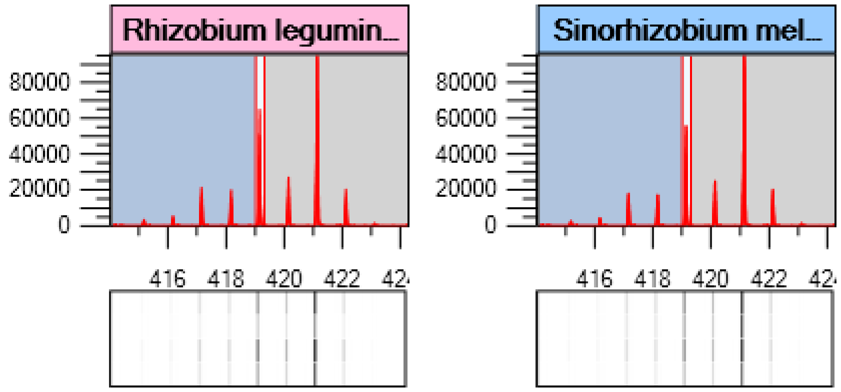

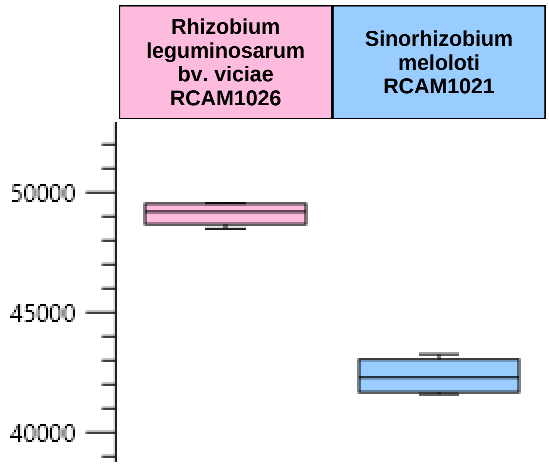

Identifier 51: m/z 421.17

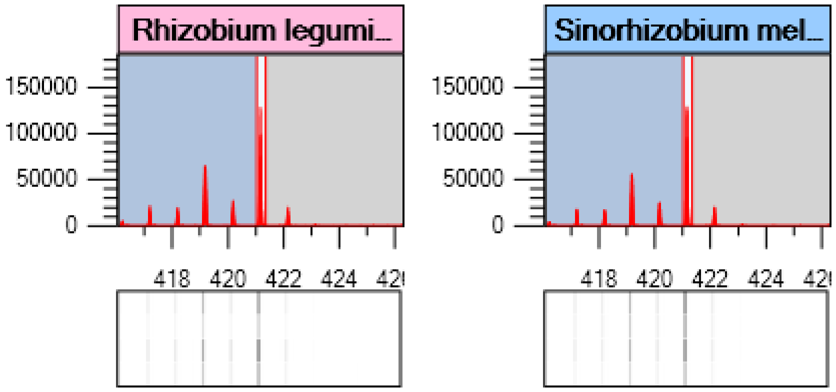

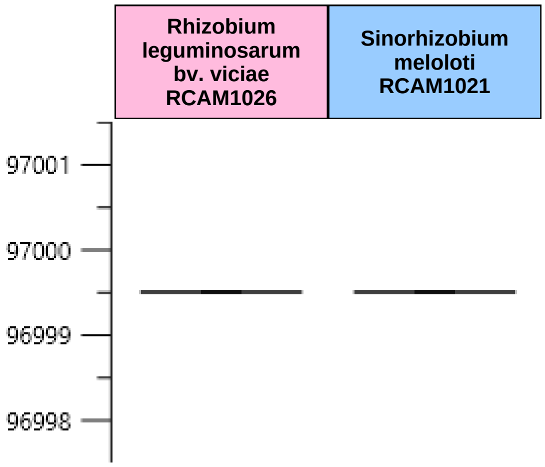

Supplement: Supplementary file 1 [file mps-03-00036-s001.zip › Supplementary information 2.pdf]
