# Supplementary material for: High-Throughput Fingerprinting of Rhizobial Free Fatty Acids by Chemical Thin-Film Deposition and Matrix-Assisted Laser Desorption/Ionization Mass Spectrometry
Source: Methods Protoc. 2020 May 4;3(2):36. doi: 10.3390/mps3020036 (PMC7359708; doi:10.3390/mps3020036)

Bacteria_AutoXecute_water-hexane_normalization by 421.17

Experiment Name: Bacteria_AutoXecute_water

Experiment Design

Group	Rhizobium leguminosarum bv. viciae RCAM1026	Sinorhizobium meloloti RCAM1021
Replicates	4	4

Spectra	Sample	Group
Rh.leg.RCAM2016_1-1-1	Rhizobium leguminosarum bv. viciae RCAM1026_1	Rhizobium leguminosarum bv. viciae RCAM1026
Rh.leg.RCAM2016_1-1-2	Rhizobium leguminosarum bv. viciae RCAM1026_1	Rhizobium leguminosarum bv. viciae RCAM1026
Rh.leg.RCAM2016_1-1-3	Rhizobium leguminosarum bv. viciae RCAM1026_1	Rhizobium leguminosarum bv. viciae RCAM1026
Rh.leg.RCAM2016_1-2-1	Rhizobium leguminosarum bv. viciae RCAM1026_1	Rhizobium leguminosarum bv. viciae RCAM1026
Rh.leg.RCAM2016_1-2-2	Rhizobium leguminosarum bv. viciae RCAM1026_1	Rhizobium leguminosarum bv. viciae RCAM1026
Rh.leg.RCAM2016_1-2-3	Rhizobium leguminosarum bv. viciae RCAM1026_1	Rhizobium leguminosarum bv. viciae RCAM1026
Rh.leg.RCAM2016_1-3-1	Rhizobium leguminosarum bv. viciae RCAM1026_1	Rhizobium leguminosarum bv. viciae RCAM1026
Rh.leg.RCAM2016_1-3-2	Rhizobium leguminosarum bv. viciae RCAM1026_1	Rhizobium leguminosarum bv. viciae RCAM1026
Rh.leg.RCAM2016_1-3-3	Rhizobium leguminosarum bv. viciae RCAM1026_1	Rhizobium leguminosarum bv. viciae RCAM1026
Rh.leg.RCAM2016_2-1-1	Rhizobium leguminosarum bv. viciae RCAM1026_2	Rhizobium leguminosarum bv. viciae RCAM1026
Rh.leg.RCAM2016_2-1-2	Rhizobium leguminosarum bv. viciae RCAM1026_2	Rhizobium leguminosarum bv. viciae RCAM1026
Rh.leg.RCAM2016_2-1-3	Rhizobium leguminosarum bv. viciae RCAM1026_2	Rhizobium leguminosarum bv. viciae RCAM1026
Rh.leg.RCAM2016_2-2-1	Rhizobium leguminosarum bv. viciae RCAM1026_2	Rhizobium leguminosarum bv. viciae RCAM1026
Rh.leg.RCAM2016_2-2-2	Rhizobium leguminosarum bv. viciae RCAM1026_2	Rhizobium leguminosarum bv. viciae RCAM1026
Rh.leg.RCAM2016_2-2-3	Rhizobium leguminosarum bv. viciae RCAM1026_2	Rhizobium leguminosarum bv. viciae RCAM1026
Rh.leg.RCAM2016_2-3-1	Rhizobium leguminosarum bv. viciae RCAM1026_2	Rhizobium leguminosarum bv. viciae RCAM1026
Rh.leg.RCAM2016_2-3-2	Rhizobium leguminosarum bv. viciae RCAM1026_2	Rhizobium leguminosarum bv. viciae RCAM1026
Rh.leg.RCAM2016_2-3-3	Rhizobium leguminosarum bv. viciae RCAM1026_2	Rhizobium leguminosarum bv. viciae RCAM1026
Rh.leg.RCAM2016_3-1-1	Rhizobium leguminosarum bv. viciae RCAM1026_3	Rhizobium leguminosarum bv. viciae RCAM1026
Rh.leg.RCAM2016_3-1-2	Rhizobium leguminosarum bv. viciae RCAM1026_3	Rhizobium leguminosarum bv. viciae RCAM1026
Rh.leg.RCAM2016_3-1-3	Rhizobium leguminosarum bv. viciae RCAM1026_3	Rhizobium leguminosarum bv. viciae RCAM1026
Rh.leg.RCAM2016_3-2-1	Rhizobium leguminosarum bv. viciae RCAM1026_3	Rhizobium leguminosarum bv. viciae RCAM1026
Rh.leg.RCAM2016_3-2-2	Rhizobium leguminosarum bv. viciae RCAM1026_3	Rhizobium leguminosarum bv. viciae RCAM1026
Rh.leg.RCAM2016_3-2-3	Rhizobium leguminosarum bv. viciae RCAM1026_3	Rhizobium leguminosarum bv. viciae RCAM1026
Rh.leg.RCAM2016_3-3-1	Rhizobium leguminosarum bv. viciae RCAM1026_3	Rhizobium leguminosarum bv. viciae RCAM1026
Rh.leg.RCAM2016_3-3-2	Rhizobium leguminosarum bv. viciae	Rhizobium leguminosarum bv. viciae

Spectra	Sample	Group
	RCAM1026_3	RCAM1026
Rh.leg.RCAM2016_3-3-3	Rhizobium leguminosarum bv. viciae RCAM1026_3	Rhizobium leguminosarum bv. viciae RCAM1026
Rh.leg.RCAM2016_4-1-1	Rhizobium leguminosarum bv. viciae RCAM1026_4	Rhizobium leguminosarum bv. viciae RCAM1026
Rh.leg.RCAM2016_4-1-2	Rhizobium leguminosarum bv. viciae RCAM1026_4	Rhizobium leguminosarum bv. viciae RCAM1026
Rh.leg.RCAM2016_4-1-3	Rhizobium leguminosarum bv. viciae RCAM1026_4	Rhizobium leguminosarum bv. viciae RCAM1026
Rh.leg.RCAM2016_4-2-1	Rhizobium leguminosarum bv. viciae RCAM1026_4	Rhizobium leguminosarum bv. viciae RCAM1026
Rh.leg.RCAM2016_4-2-2	Rhizobium leguminosarum bv. viciae RCAM1026_4	Rhizobium leguminosarum bv. viciae RCAM1026
Rh.leg.RCAM2016_4-2-3	Rhizobium leguminosarum bv. viciae RCAM1026_4	Rhizobium leguminosarum bv. viciae RCAM1026
Rh.leg.RCAM2016_4-3-1	Rhizobium leguminosarum bv. viciae RCAM1026_4	Rhizobium leguminosarum bv. viciae RCAM1026
Rh.leg.RCAM2016_4-3-2	Rhizobium leguminosarum bv. viciae RCAM1026_4	Rhizobium leguminosarum bv. viciae RCAM1026
Rh.leg.RCAM2016_4-3-3	Rhizobium leguminosarum bv. viciae RCAM1026_4	Rhizobium leguminosarum bv. viciae RCAM1026
Si.mel.RCAM1021_1-1-1	Sinorhizobium meloloti RCAM1021_1	Sinorhizobium meloloti RCAM1021
Si.mel.RCAM1021_1-1-2	Sinorhizobium meloloti RCAM1021_1	Sinorhizobium meloloti RCAM1021
Si.mel.RCAM1021_1-1-3	Sinorhizobium meloloti RCAM1021_1	Sinorhizobium meloloti RCAM1021
Si.mel.RCAM1021_1-2-1	Sinorhizobium meloloti RCAM1021_1	Sinorhizobium meloloti RCAM1021
Si.mel.RCAM1021_1-2-2	Sinorhizobium meloloti RCAM1021_1	Sinorhizobium meloloti RCAM1021
Si.mel.RCAM1021_1-2-3	Sinorhizobium meloloti RCAM1021_1	Sinorhizobium meloloti RCAM1021
Si.mel.RCAM1021_1-3-1	Sinorhizobium meloloti RCAM1021_1	Sinorhizobium meloloti RCAM1021
Si.mel.RCAM1021_1-3-2	Sinorhizobium meloloti RCAM1021_1	Sinorhizobium meloloti RCAM1021
Si.mel.RCAM1021_1-3-3	Sinorhizobium meloloti RCAM1021_1	Sinorhizobium meloloti RCAM1021
Si.mel.RCAM1021_2-1-1	Sinorhizobium meloloti RCAM1021_2	Sinorhizobium meloloti RCAM1021
Si.mel.RCAM1021_2-1-2	Sinorhizobium meloloti RCAM1021_2	Sinorhizobium meloloti RCAM1021
Si.mel.RCAM1021_2-1-3	Sinorhizobium meloloti RCAM1021_2	Sinorhizobium meloloti RCAM1021
Si.mel.RCAM1021_2-2-1	Sinorhizobium meloloti RCAM1021_2	Sinorhizobium meloloti RCAM1021
Si.mel.RCAM1021_2-2-2	Sinorhizobium meloloti RCAM1021_2	Sinorhizobium meloloti RCAM1021
Si.mel.RCAM1021_2-2-3	Sinorhizobium meloloti RCAM1021_2	Sinorhizobium meloloti RCAM1021
Si.mel.RCAM1021_2-3-1	Sinorhizobium meloloti RCAM1021_2	Sinorhizobium meloloti RCAM1021
Si.mel.RCAM1021_2-3-2	Sinorhizobium meloloti RCAM1021_2	Sinorhizobium meloloti RCAM1021
Si.mel.RCAM1021_2-3-3	Sinorhizobium meloloti RCAM1021_2	Sinorhizobium meloloti RCAM1021
Si.mel.RCAM1021_3-1-1	Sinorhizobium meloloti RCAM1021_3	Sinorhizobium meloloti RCAM1021
Si.mel.RCAM1021_3-1-2	Sinorhizobium meloloti RCAM1021_3	Sinorhizobium meloloti RCAM1021
Si.mel.RCAM1021_3-1-3	Sinorhizobium meloloti RCAM1021_3	Sinorhizobium meloloti RCAM1021
Si.mel.RCAM1021_3-2-1	Sinorhizobium meloloti RCAM1021_3	Sinorhizobium meloloti RCAM1021
Si.mel.RCAM1021_3-2-2	Sinorhizobium meloloti RCAM1021_3	Sinorhizobium meloloti RCAM1021
Si.mel.RCAM1021_3-2-3	Sinorhizobium meloloti RCAM1021_3	Sinorhizobium meloloti RCAM1021
Si.mel.RCAM1021_3-3-1	Sinorhizobium meloloti RCAM1021_3	Sinorhizobium meloloti RCAM1021
Si.mel.RCAM1021_3-3-2	Sinorhizobium meloloti RCAM1021_3	Sinorhizobium meloloti RCAM1021
Si.mel.RCAM1021_3-3-3	Sinorhizobium meloloti RCAM1021_3	Sinorhizobium meloloti RCAM1021
Si.mel.RCAM1021_4-1-1	Sinorhizobium meloloti RCAM1021_4	Sinorhizobium meloloti RCAM1021
Si.mel.RCAM1021_4-1-2	Sinorhizobium meloloti RCAM1021_4	Sinorhizobium meloloti RCAM1021
Si.mel.RCAM1021_4-1-3	Sinorhizobium meloloti RCAM1021_4	Sinorhizobium meloloti RCAM1021
Si.mel.RCAM1021_4-2-1	Sinorhizobium meloloti RCAM1021_4	Sinorhizobium meloloti RCAM1021
Si.mel.RCAM1021_4-2-2	Sinorhizobium meloloti RCAM1021_4	Sinorhizobium meloloti RCAM1021
Si.mel.RCAM1021_4-2-3	Sinorhizobium meloloti RCAM1021_4	Sinorhizobium meloloti RCAM1021
Si.mel.RCAM1021_4-3-1	Sinorhizobium meloloti RCAM1021_4	Sinorhizobium meloloti RCAM1021
Si.mel.RCAM1021_4-3-2	Sinorhizobium meloloti RCAM1021_4	Sinorhizobium meloloti RCAM1021
Si.mel.RCAM1021_4-3-3	Sinorhizobium meloloti RCAM1021_4	Sinorhizobium meloloti RCAM1021

Statistics Measurement: Normalized Peak Height

Normalization Method: Single Peak Height 421.17 m/z

Peaks

#	m/z	Fold	Anova (p)	Tags
3	363.10	1.09	0.015	
4	365.11	1.17	7.55E-05	
10	377.11	1.10	0.0275	
12	379.12	1.14	0.00342	
23	391.12	1.06	0.00172	
25	393.14	1.00	1	
43	417.14	1.04	0.173	
47	419.15	1.04	0.187	
50	421.17	1.00	1	

Peaks : Average Normalized Peak Heights

#	Rhizobium leguminosarum bv. viciae RCAM1026	Sinorhizobium meloloti RCAM1021
3	1387.725	1516.819
4	4651.020	5425.327
10	2067.367	2276.266
12	7042.831	8008.104
23	2.596e+004	2.760e+004
25	4.136e+004	4.143e+004
43	2.745e+004	2.634e+004
47	8.030e+004	7.737e+004
50	1.274e+005	1.274e+005

Identifier 3: m/z 363.10

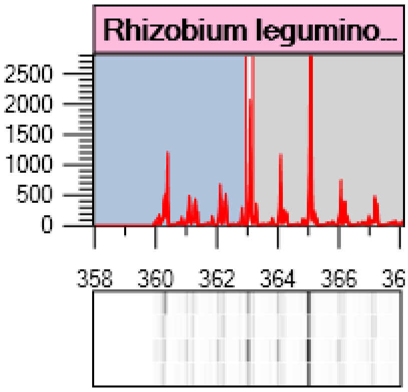

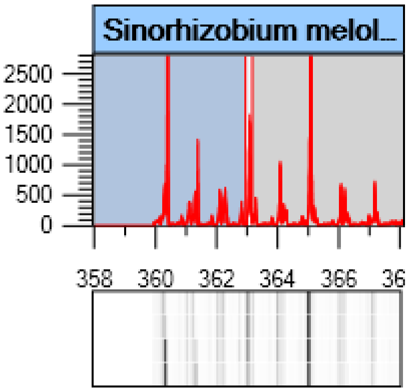

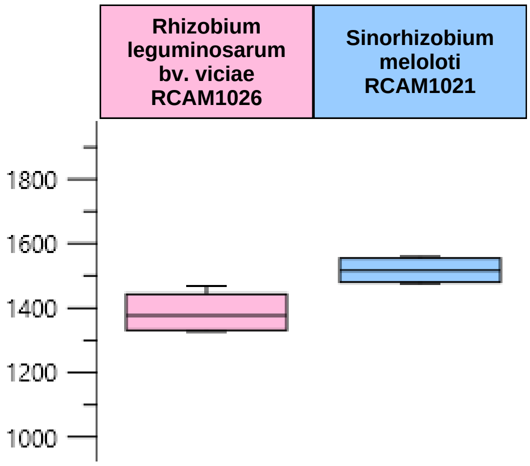

Identifier 4: m/z 365.11

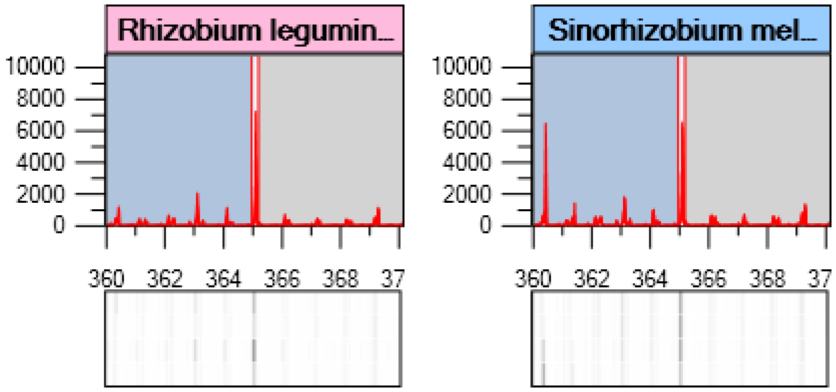

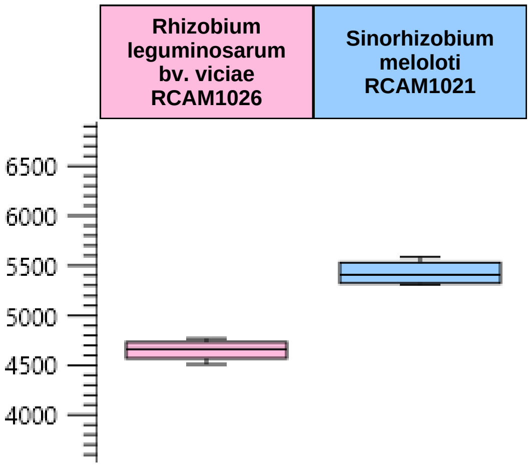

nonlinear dynamics

Identifier 10: m/z 377.11

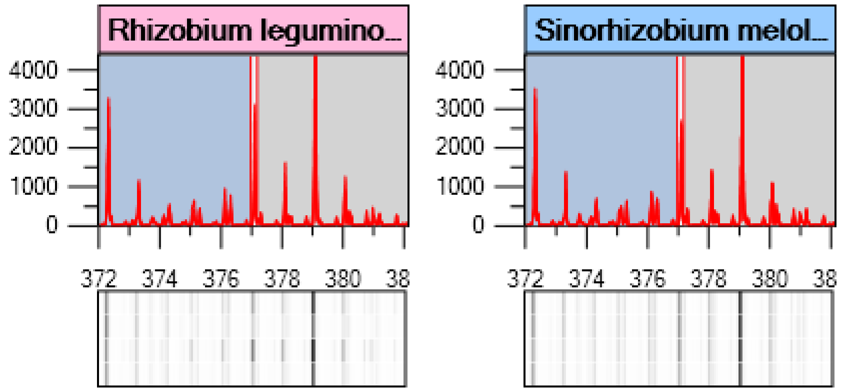

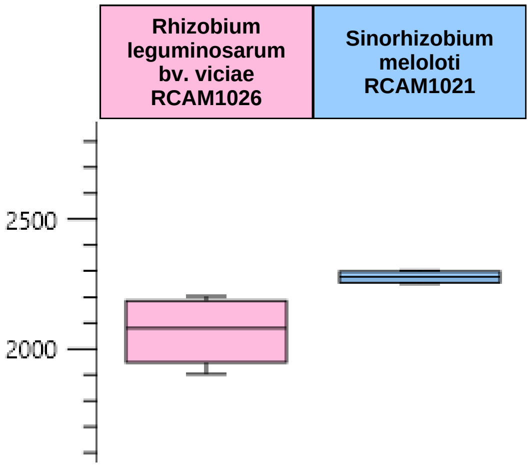

Identifier 12: m/z 379.12

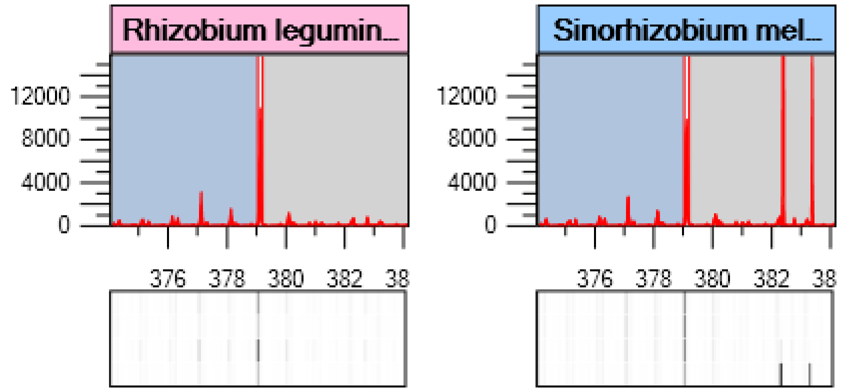

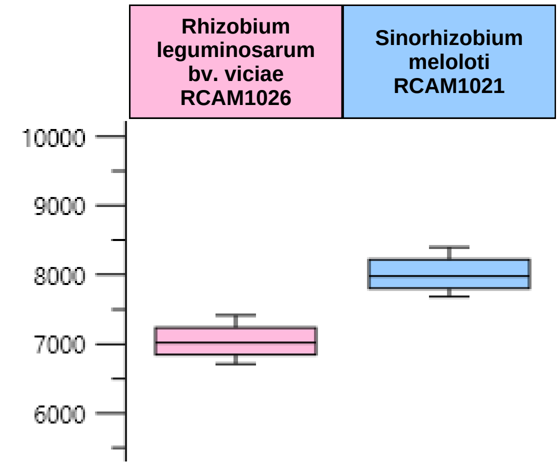

Identifier 23: m/z 391.12

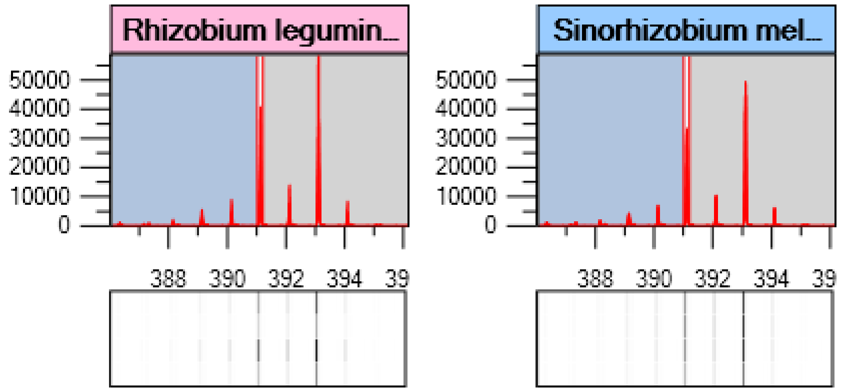

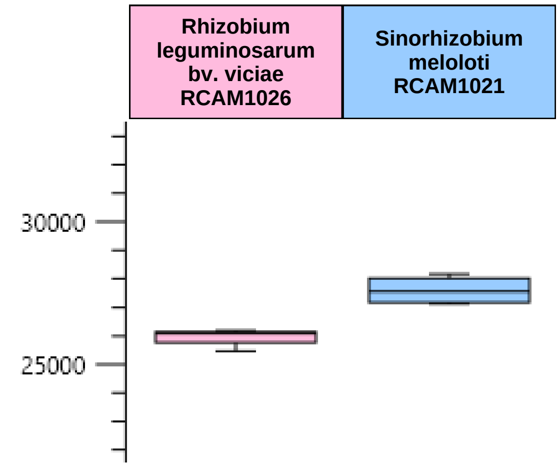

Identifier 25: m/z 393.14

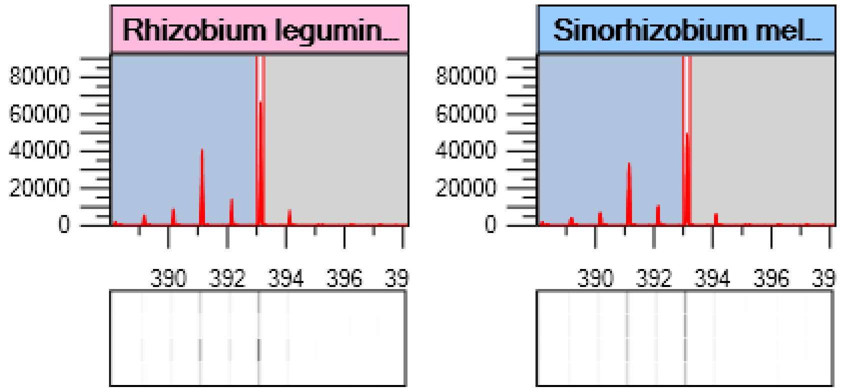

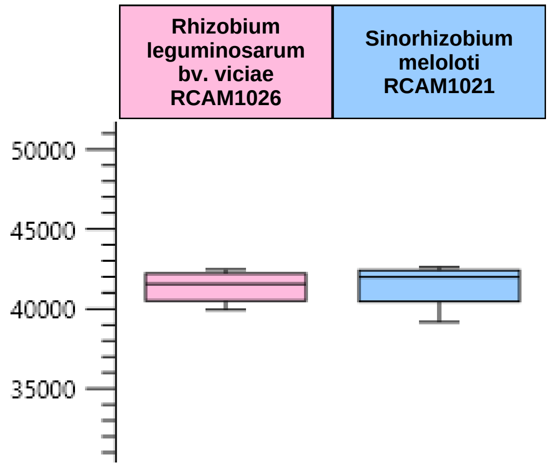

Identifier 43: m/z 417.14

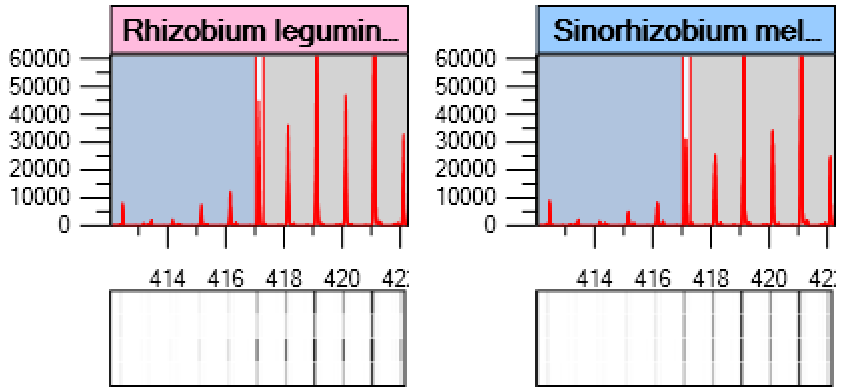

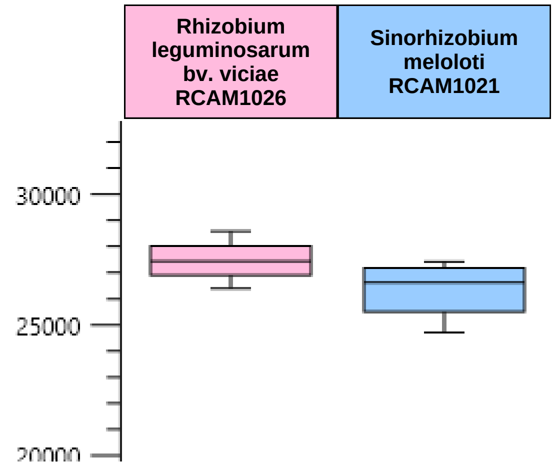

Identifier 47: m/z 419.15

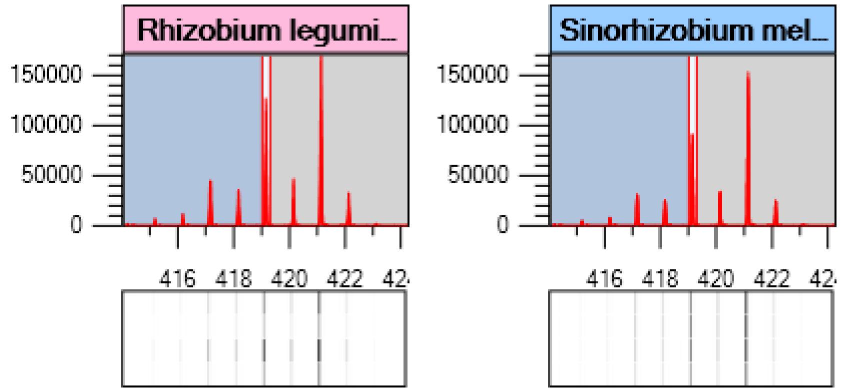

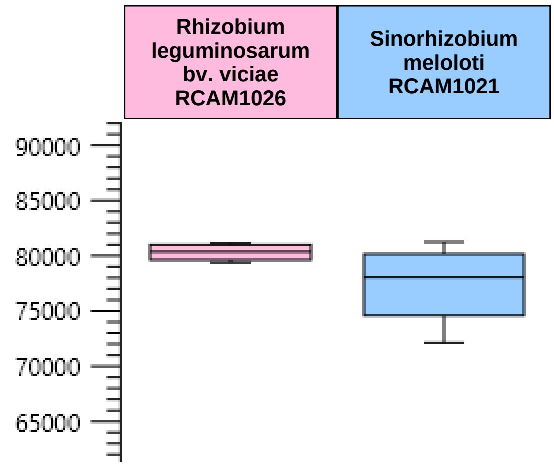

Identifier 50: m/z 421.17

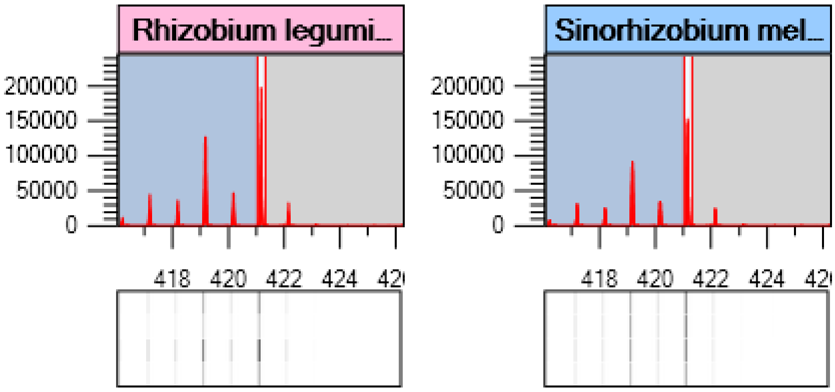

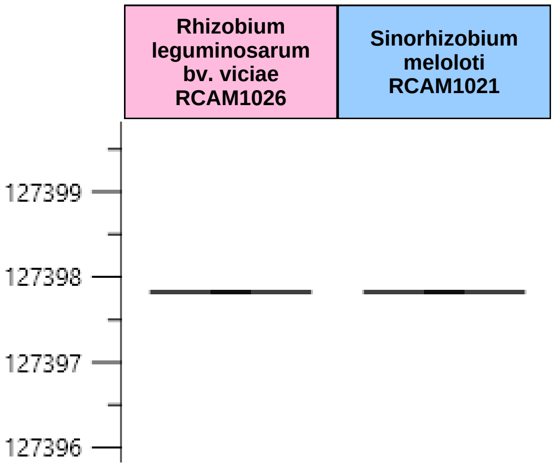

Supplement: Supplementary file 1 [file mps-03-00036-s001.zip › Supplementary information 3.pdf]
